# Supplementary material for: Multiscale predictive modeling robustly improves the accuracy of pseudo-prospective seizure forecasting in drug-resistant epilepsy
Source: bioRxiv. 2025 Sep 29:2025.09.27.678967. Originally published 2025 Sep 28. Preprint. [Version 2] doi: 10.1101/2025.09.27.678967 (PMC12485719; doi:10.1101/2025.09.27.678967)
Supplement: 1 [file NIHPP2025.09.27.678967V2-supplement-1.pdf]

bioRxiv preprint doi: https://doi.org/10.1101/2025.09.27.678967; this version posted September 29, 2025. The copyright holder for this preprint (which was not certified by peer review) is the author/funder, who has granted bioRxiv a license to display the preprint in perpetuity. It is made available under aCC-BY 4.0 International license.

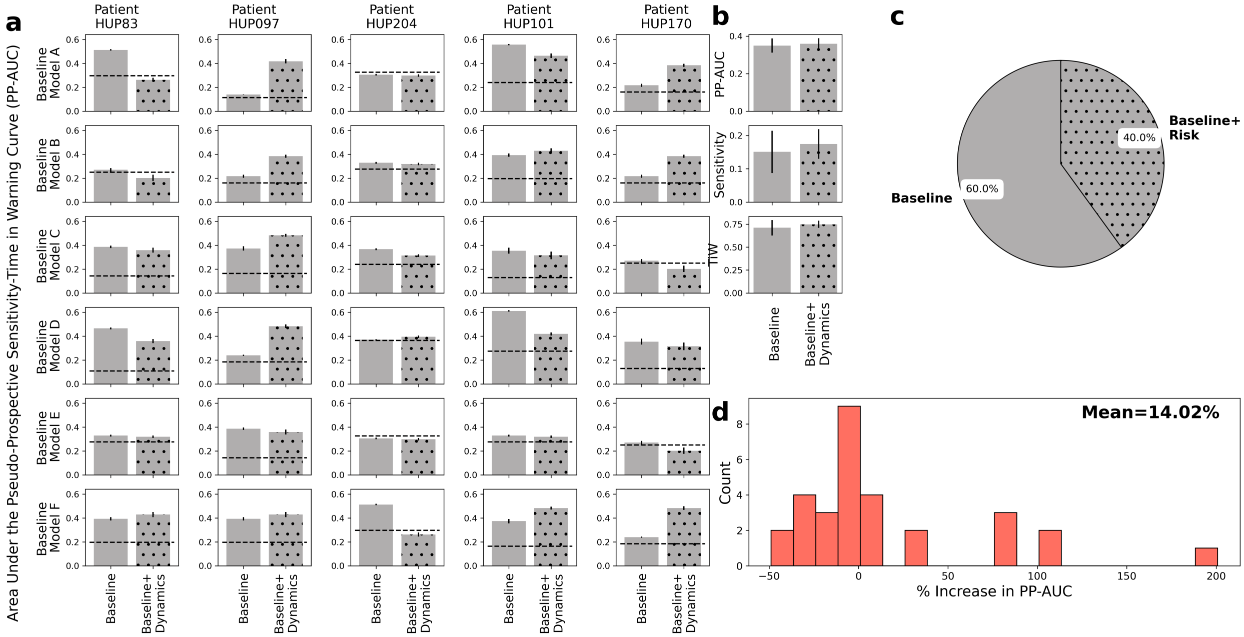

Supplementary Figure 1: **Relative improvements in pseudo-prospective forecasting accuracy using risk dynamical modeling alone.** Panels parallel those in Figures 5 and 7. Note the lack of color coding (corresponding to different feature dynamical models which are absent here) and the use of dotted hatches for risk-augmented models.
